# Development of a Robust Canine Welfare Assessment Protocol for Use in Dog (Canis Familiaris) Catch-Neuter-Return (CNR) Programmes

**DOI:** 10.3390/ani9080564

**Published:** 2019-08-16

**Authors:** Heather Bacon, Hayley Walters, Vlad Vancia, Louise Connelly, Natalie Waran

**Affiliations:** 1 Jeanne Marchig International Centre for Animal Welfare Education, Royal (Dick) School of Veterinary Studies, University of Edinburgh, Edinburgh EH25 9RG, UK; 2 The Romanian Society for Anthrozoology, Cluj 400372, Romania; 3 Faculty of Education, Humanities and Health Sciences, Eastern Institute of Technology, 501 Gloucester St, Taradale, Napier 4112, New Zealand

**Keywords:** animal welfare, assessment, canis familiaris, dog, catch-neuter-return, CNR, Trap-neuter-return

## Abstract

**Simple Summary:**

This paper describes the development of a robust composite welfare assessment tool to evaluate the welfare of free-roaming dogs passing through surgical sterilisation or catch-neuter-return (CNR) programmes. Catch-Neuter-Return programmes are frequently employed by governments and animal welfare charities to control the rising population of free-roaming dogs. Due to the focus on dog population control, individual dog welfare may be compromised through the CNR process, which comprises necessary stressors and harms, including capture, transport, surgery, and social disruption. Using a combination of a modified Delphi analysis and a Hazard Identification and a Critical Control Point analysis, this project described potential hazards to dog welfare during the CNR process and identified critical points where dog welfare could be assessed, and mitigations put in place to improve dog welfare throughout the CNR process. This project resulted in the development of a composite dog welfare assessment tool that will allow the robust assessment of the welfare of free-roaming dogs in CNR programmes and allow CNR projects to benchmark and mitigate hazards to dog welfare.

**Abstract:**

The aim of this study was to develop a welfare assessment tool based on objective, reliable and relevant measures to be applied to individual dogs as they underwent a Catch-Neuter-Return (CNR) programme. A modified Delphi method and Hazard Analysis and Critical Control Points (HACCP) approach was used to develop the composite canine welfare assessment protocol, comprising both animal-based and resource-based measures. This draft welfare assessment protocol was then trialed and refined in existing CNR programmes to identify key control points where individual dog welfare may be moderately or significantly compromised in the CNR process. The results show that animal-based welfare indicators, e.g., pain behaviours, which provide a more direct indication of an animal’s welfare state, require training and skill to recognise, whilst resource-based indicators are simple to measure but act only as indirect measures of welfare. We concluded that whilst CNR projects can potentially improve the health and welfare of free-roaming dogs in the long-term, the risk of short-term welfare harms during the CNR process is high. Thus, it is essential for staff involved in dog population management programmes to assess the welfare state of dogs in CNR and take remedial action to safeguard individual dog welfare.

## 1. Introduction

Catch Neuter Return (CNR) programmes are often advocated by governments and animal welfare groups as being an appropriate solution to the control of dog populations [1,2,3,4]. CNR is considered by animal welfare charities, academics and the World Organisation for Animal Health (OIE) to be an essential tool in the control of dog populations, zoonoses and human-dog conflicts [5,6,7,8]. The ubiquitous nature of CNR, its application by leading animal welfare organisations, and the poor welfare implications of alternative dog population control measures, all contribute to the perception of CNR as a positive welfare intervention. CNR-type programmes are more likely to be utilised in more developed countries [9], where one may expect standards of veterinary training and dog handling to be higher. However, the variety of techniques used in CNR projects combined with the focus on population control, may result in poor welfare for individual dogs within the programme [10,11].

The Hazard Analysis and Critical Control Point (HACCP) approach is used in a variety of industries to identify key stages within a process where potential hazards to a process may occur [12,13,14]. These stages are referred to as Critical Control Points (CCPs). The identification of potential hazards and CCPs allows for the development of evaluation and mitigation at these key points. The HACCP approach has been widely employed in the food security process to identify and rectify possible hazards to food safety and has been adapted for welfare assessment in the livestock industry [14,15,16,17]. Hazards were refined through a modified Delphi process. The Delphi process is a consensus stakeholder analysis [18] and has been utilised previously to identify hazards to animal welfare across a wide range of species [18,19,20,21]. Previous Delphi analyses in dogs and other species have relied on consensus from as few as seven [21] to as many as 154 [18] experts. In this project, we employed the HACCP approach for the first time to identify hazards that may compromise canine welfare in CNR programmes.

## 2. Materials and Methods

This was a purely observational study and no animals were used or harmed in this study. Ethical review was given by the Royal (Dick) school of veterinary studies Survey overview group ethics committee. No ethical review number was assigned. 

The HACCP process is well documented and the approach as applied to this project is summarised in Table 1.

### 2.1. Defining Project Outcomes and the CNR Process

**Figure 1:**
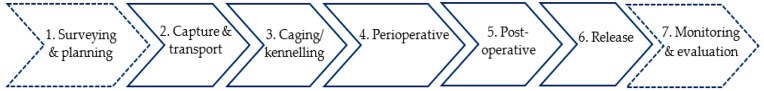
Flow diagram outlining the seven primary stages of CNR. Solid outline = stages that were determined to directly impact upon dog welfare.

The research team was assembled and ethical approval was obtained via the Veterinary and Human Ethical Review Committees at the University of Edinburgh’s Royal (Dick) School of veterinary studies. The product—a robust canine welfare audit—was described: the key features identified were that it needed to be practical, economical and reliable, and it should identify moderate to significant welfare hazards within the CNR process, recognising that milder welfare insults are inevitable during a CNR intervention comprising of capture, transport and surgery. A flow diagram considered all aspects of the CNR process and identified seven primary stages of CNR (Figure 1), five of these stages were considered to impact dog welfare directly and were thus considered in this project. The seven stages identified were 1. Surveying and planning—the logistical process and demographic survey that should be completed prior to undertaking a CNR project. As this stage is non-invasive to dogs, it was not considered to directly impact dog welfare, although it is essential for the success of a CNR programme. 2. Capture and transport—the removal of free-roaming dogs from the street and their transportation to the clinical site. 3. Caging/kenneling—the confinement of dogs at the clinical site prior to surgical intervention. 4. Perioperative—the time frame spanning dogs being removed from their cage/kennelling area and undergoing sedation to them being returned to their cage/kennelling area post-surgery. 5. Post-operative—The period of time between dogs returning to their cage/kennelling area after surgery and being released. 6. Release—The processes including handling, transport and return to the release site. 7. Monitoring and Evaluation—the process of data analysis, follow-up surveying and evaluation of impact. As this stage is non-invasive to dogs, it was not considered to directly impact dog welfare, although it is essential for the success of a CNR programme.

### 2.2. Hazard Identification

Hazard identification was achieved through a sequential process of first reviewing the literature on existing dog welfare indicators, and secondly, conducting a two-stage modified Delphi process. A thorough review of the existing published and grey literature relating to dog population management, neutering, kenneling, veterinary care and canine welfare assessment was completed using Web of Science, Google scholar and PubMed, in addition to contacting relevant organisations working in the field for unpublished guidelines and protocols. This search identified behavioural, physiological and resource-based indicators that may be employed to identify hazards to canine welfare. A questionnaire of these canine welfare indicators developed from the literature review and from practical experiences within the research team was formulated. Each indicator on the list was assigned to one of the five stages of the CNR process based on the authors’ experience and context from the literature. Some indicators were modified slightly to accommodate practical realities of CNR. For example, the anesthetic mortality in dogs is generally reported as 0.03–0.12% [22,23,24], however we initially set a threshold of mortality at 2%, recognizing that pre-operative health assessment and prophylactic medical care was likely to be much more limited in street dogs and thus, the risks of unanticipated peri-operative deaths much higher. Due to the field trials, this threshold was later revised and lowered. Similarly, the threshold for surgical time was set at 90 min, based on the durations reported in the literature at which infection risks significantly increased [25]. Some indicators were identified as being potentially useful in more than one stage.

### 2.3. The Expert Panel

A list of experienced stakeholders was developed by utilising a matrix approach to ensure that expertise across a breadth of dog management experience was sought. The categories of expertise utilised in the matrix were ‘clinical CNR’, ‘dog behaviour, welfare, or pain assessment’, ‘dog population management’, and ‘dog kennelling and housing’. A maximum variation purposive sample was selected from veterinary organisations and non-governmental organisations (NGOs) working in the fields of canine medicine, CNR, or animal behaviour. A maximum purposive sample is a non-probability sampling technique that seeks to ensure a relevant sample of expertise is recruited. A minimum of five experts were recruited to each category of the matrix, resulting in 26 stakeholders. Experts were recruited based on their published work and experience in the field of CNR. Each expert was contacted confidentially with an introduction to the project and the option to decline involvement. Following this, one respondent withdrew from the process, leaving 25 participants.

### 2.4. The Modified Delphi Process

A two-stage process was employed. In the first stage of the Delphi analysis, experts were provided with the questionnaire, including the list of potential behavioural, physiological and resource-based welfare indicators for each stage of the CNR process and asked to contribute to the list with any further suggested indicators or hazards to dog welfare in CNR, based on their own experience. 

The expert panel (*n* = 25) was then asked to score each indicator for practicality (how easily it could be utilised in a field CNR situation), economics (its value for money), and validity (how accurately it represented the dog’s welfare state). Each indicator received 1 point when scored by the respondents for any of the criteria ‘practical’, ‘economical’, or valid’ (maximum of 3 points per indicator per respondent). 

The complete list of indicators was then re-sent to the 25 original Delphi participants in a second round where participants were asked to score each indicator/hazard according to the level of welfare compromise it represented on a scale of mild-moderate-severe. Mild was defined as ‘short-term or minor physical or mental welfare compromise with which the dog can cope and adapt’. Moderate was defined as ‘a welfare compromise which is likely to cause behavioural or mental distress or physical pain or injury, but which does not result in long-term effects’. Severe was defined as ‘a level of welfare compromise which is likely to cause long-term physical discomfort/impairment or on-going mental/behavioural distress, or affect the dog’s survivability’.

Each indicator/hazard received 1 point when scored by the Delphi respondents for either of the criteria ‘moderate’ or ‘severe’ welfare compromise (maximum of 1 point per indicator per respondent). Moderate and severe indicators received the same score, as we were simply interested in distinguishing them from ‘mild’ indicators, not attempting to rank these indicators in any way. Indicators scored as ‘mild’ welfare compromise received zero points during this round, as the research team considered that mild welfare compromise was inevitable during the handling and surgery of dogs, and by definition, a mild welfare insult was one that dogs would be able to cope with during the CNR process. 

The indicators and hazards were ranked according to response scores. There is a lack of accepted guidance on the level of group agreement required in this process e.g., 51% [21,26], 80% [27]. Whaytt et al. devised the process of ranking Delphi responses by calculating the percentage of the maximum response score (%MRS) for each indicator [18] and this approach was also used in this project. We calculated the %MRS for each indicator across the categories of practicality, reliability, economy and welfare significance. %MRS ranged from 2.2% (microchipping) to 100% (post-operative pain behaviour). As mentioned above, MRS > 50% is often selected as a threshold for group agreement, however, in this project, because all the indicators were derived either from the existing evidence in the literature or from expert consensus, they were considered to be valid indicators of moderate–severe welfare problems and a low threshold of 30% MRS was set with all indicators scoring over this, retained in the study in order to allow a maximum number of indicators to be trialed in the field. 

### 2.5. Inter-Observer and Intra-Observer Reliability Testing

The assessment of some welfare indicators and hazards was very straightforward, e.g., ‘dog offered drinking water prior to release’ or ‘dog provided with bedding in kennel’ was directly observable and not subject to interpretation, but some of the indicators listed were potentially open to interpretation. In particular, the behavioural or human–dog-interaction indicators had the potential for subjectivity in assessment, e.g., dog shows signs of ‘post-operative pain’, or ‘acceptable handling technique used’ may be assessed differently by different observers. To ensure inter-observer reliability in the field tests, two researchers with extensive practical experience in dog behaviour, handling and clinical care, performed inter- and intra-observer reliability testing, as described in [28] to ensure that they could consistently recognise and evaluate practices in free-roaming dog capture and handling, and in dog behavioural responses.

### 2.6. Field Trial A—On-Site Confirmation and Initial Evaluation of Indicators

Once it was established that the welfare indicators/hazards to welfare could be reliably identified by the research team, field sites to trial the composite welfare assessment were identified. The primary characteristics of the two field trial sites are shown in Table 2, but the sites were anonymized as trial site A and trial site B.

Field trial A consisted of a four-day test in which a convenience sample of 12 street dogs was followed throughout the entire process of a well-established CNR programme, with an additional 14 dogs observed for sections of the CNR process. Each of the 53 indicators on the draft audit was applied to the test dogs as they went through the CNR process. 

Based on the trialing of the draft audit at the CNR programme, a number of suggested indicators were removed from the draft audit as they were found to be impractical, unreliable or not financially possible, e.g., heart rate variability, lymphocyte: neutrophil ratio. 

Additionally, some indicators that had proven useful in more than one category were duplicated across categories where appropriate. One high-scoring indicator (pre-existing injury at time of capture 82% MRS) was removed from the study, as it was not thought to accurately reflect a welfare problem that was controllable within the scope of the CNR process, but instead reflected a pre-existing welfare problem. It also became apparent that some of the record-based and resource-based indicators, e.g., mortality rate, administration of vaccinations, and the presence of a crash kit, were either not related to a specific stage within the CNR process or not representative of individual dog welfare, but were instead programmatic indicators of good practice, therefore, a separate category was created on the draft audit for these indicators. 

Based on this first field test, some of the descriptors of the CCPs were modified to minimise the potential for variation in interpretation by an observer. For example ‘excessive force in capture’ was changed to ‘use of capture equipment, e.g., loop pole, net or neck grasper’, as this equipment was consistently presented by Delphi survey respondents as a reason for unacceptable welfare in capture and handling, and the use of these tools is a more objective measurement than attempting to determine ‘excessive’ force. Similarly, ‘use of excessive force in handling’ (a negative welfare indicator) was modified to ‘Handler checks dog’s response before handling’ (a positive indicator), as it was felt that ‘excessive force’ was too subjective a term and would create variation in assessment. If the handler checks a dog’s response before handling by allowing it to sniff at the handler or by stroking it and gauging its response, this represents an understanding of tailoring animal handling techniques to an individual dog and would be a positive indicator.

A previous assessment of dog behaviour using video trials [28] had demonstrated that determining very specific welfare states, e.g., ‘calm-alert’ vs. ‘calm-relaxed’ was too difficult to be carried out reliably, and even determining ‘positive’ and ‘negative’ welfare states based on a dog’s demeanour and behaviours generated variation in responses [28]. However, it was decided to retain some behavioural welfare indicators within the draft welfare audit, as animal-based indicators were recognised to be more robust than resource-based indicators [18,19]. These indicators included ‘anxiety/fear behaviour’, ‘aggressive behaviour’, ‘escape behaviour’, ‘exploratory behaviour’, and ‘pain behaviour’ and their retention was based on their high %MRS and thus, expert assessment of their significance to dog welfare. 

### 2.7. Defining Critical Control Points (Ccps)

**Figure 2:**
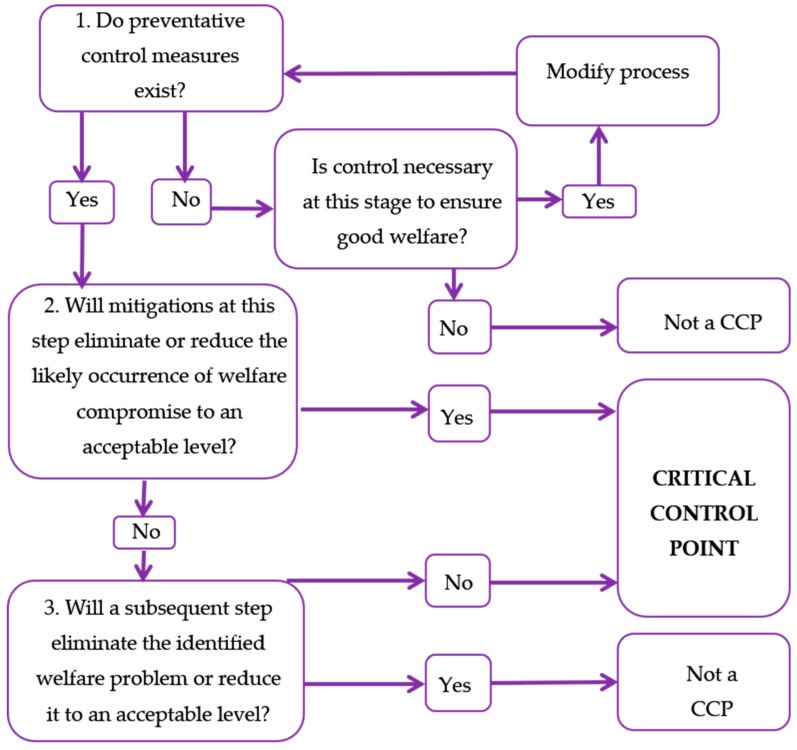
Critical control point algorithm (adapted from [31]).

The HACCP process relies on establishing Critical Control Points (CCPs) once all potential hazards are identified [29,30]. Each welfare indicator, representing a potential dog welfare hazard, generated through the HACCP process, was evaluated as a CCP, against a modified CCP algorithm (Figure 2) to determine its appropriateness as a critical control point. Based on this assessment, 46 reliable, economical and practical indicators of moderate to severe welfare compromise were included in the second draft dog welfare audit, and, as some indicators were duplicated across stages in the CNR process, e.g., escape behaviour, provision of water etc., this resulted in 66 potential CCPs or welfare evaluation points. The second draft of the dog welfare audit was piloted at a second field trial (field trial B).

### 2.8. Field Trial B—Inter-Observer Reliability Testing

The inter-observer reliability testing process was repeated with a three-observer team, including the two original observers who had already demonstrated consistency prior to field trial B as the behaviour-based CCPs included on the welfare audit had been edited and revised. It was important that despite these revisions, the behaviours they represented be consistently recognised by all three observers participating in field trial B. The results of a survey of CNR professionals in assessing dog behaviour had indicated that training in consistently identifying some or all of these behaviours is necessary even amongst experienced professionals working with dogs [28]. Each observer reviewed the behavioural indicators of emotional state as described by Tod et al. [32].

Inter-observer reliability was then tested between observers using ten dogs at a local shelter and veterinary hospital and a range of 28 video clips. Each observer viewed the behaviours shown by the observed dogs and individually recorded whether any of the five behavioural welfare indicators (aggression, escape, exploratory, fear, pain) included in the draft welfare audit and based on the descriptors published by Tod et al. was shown by each dog. Consensus in responses was evaluated using Fleiss’ Kappa attribute agreement analysis in the Minitab 17 statistical software.

### 2.9. Field Trial B—Verification of the Refined Welfare Audit

The utility of the welfare audit was verified at a second CNR project field trial B in a different geographic location and with a different capture technique, veterinary capacity and return approach to ensure its flexibility across different programmatic structures. Three observers followed the dogs for five days, observing all stages of the CNR programme. Data were recorded using the welfare audit in Microsoft excel, Microsoft, Manchester, UK format on Psion Workabout Pro 3, Psion PLC, London, UK. In this field trial, a convenience sample of 53 dogs were followed from capture through to release or occasionally, euthanasia on humane grounds, and 88 additional dogs were evaluated during sections of the CNR process. 

Following this final field trial, the CCPs on the draft audit were revised once more, for example programmatic indicators such as the >2% mortality rate were discussed with the projects involved in the trials and the consensus was that this threshold was too lenient and thus it was lowered to >1%, still higher than the peri-operative mortality rate that might be anticipated in a more controlled surgical environment, but a realistic threshold for a CNR project dealing with dogs of unknown health status. Thus, the CNR dog welfare audit was finalised with expert input from the field trial projects. To enhance accessibility, this audit is available as a free mobile phone application (Dog Welfare Assessment app) on android and apple platforms [33].

## 3. Results

### 3.1. Delphi Results

We received 13 expert responses (52% response rate) to round one of the modified Delphi process, resulting in 96 behavioural, physical, physiological and resource-based indicators of welfare or potential hazards to welfare. We received 11 expert responses (44% response rate) to round two of the modified Delphi process. The ranking of the indicators above a 30% MRS resulted in a list of 53 resource-based and animal-based (physical, physiological and behavioural) welfare indicators and hazards to be trialed as a draft audit (Appendix A).

### 3.2. Field Trial A—Inter- and Intra-Observer Reliability Test

It was essential that the research team be able to reliably recognise the animal-based indicators used in the welfare audit. Inter- and intra-observer reliability tests were evaluated for levels of agreement using a Fleiss Kappa test. These results are reported in [28].

### 3.3. Field Trial B—Inter-Observer Reliability Test Results

The second round of inter-observer reliability testing prior to Field Test B comprised three observers evaluating five behavioural CCPs using video footage and ten veterinary inpatient and shelter dogs. The test demonstrated agreement ranging from substantial to nearly perfect [34] between the three observers (Table 3).

### 3.4. The Final Canine Welfare Audit

The final canine welfare audit is shown below (Table 4). It provides a mechanism for identifying gaps in care currently delivered to dogs going through a CNR programme and the quantification of the problems identified as a percentage of the total population of dogs. By using the welfare audit, individual CNR programmes may be systematically reviewed and critical control points currently not being considered may be identified in order to improve canine welfare within the programme.

### 3.5. Primary CCPs Detected

Table 5 shows the most common problems detected at the two CNR projects participating in the study. The two projects are not directly comparable, as the indicators evaluated were refined and modified between the two field trials. However, the results do demonstrate the most common issues that arose within each of the two projects. The absence of a positively valenced CCP indicates a welfare concern and conversely, the presence of a negatively valenced CCP also indicates a welfare concern. The most common issue that arose in both projects was the recording of pain behaviours in either 56% (field site A) or 60% (field site B) of dogs, despite both projects administering a non-steroidal anti-inflammatory drug (NSAID) analgesic prior to surgery.

## 4. Discussion

This project applied the HACCP principles, a review of the literature, the modified Delphi method, and practical field trials to develop a robust, practical and economical canine welfare audit. The welfare audit proved to be a useful tool to assess dog welfare in the field. All three observers felt that it comprehensively evaluated all stages of CNR and flagged key areas of concern not previously identified by staff within the CNR programme. Different welfare problems were detected in CNR project B when compared to CNR project A, where many of the same indicators were used across both assessments, demonstrating the flexibility of the welfare audit in identifying a broad range of welfare problems across two CNR programmes with very different techniques.

Selecting the indicators used within the audit was a multiple-stage process founded in the literature and with expert input through the modified Delphi process. The literature was useful for generating many of the potential hazards/welfare indicators and for providing thresholds for surgical time and mortality rates [22,25]. This was a useful step as it generated potential indicators that had not been identified in the literature such as ‘ear tag placed’. This example highlights the challenges of weighing up the cost-benefits within CNR programmes—all the dogs that pass through CNR programmes should be identified in order to prevent repeat capture at a later date and whilst the welfare implications of ear tagging may not be immediately obvious, it was suggested that ear tag placement could have longer-term detrimental impacts on welfare through the risks of chronic infection at the site of the tag or the risk of trauma should the tag be snagged. Alternative methods of marking neutered dogs such as ear-tipping were instead viewed as being less of a welfare risk. 

Whilst acceptable and unacceptable limits are traditionally set for critical control points (CCPs), in order to maximise the practicality of the welfare audit in the field, a present/absent approach to CCPs was considered to be most appropriate for this assessment. For many of the CCPs, setting limits was not actually possible or practical, e.g., surgery was either performed by a student or an untrained vet or it was performed by an experienced vet, and drinking water was provided or it was not. In this study, any level of an identified welfare hazard would indicate an unacceptable deterioration in welfare state, as all suggested CCPs represented a moderate or severe welfare compromise, as ranked by the Delphi participants, therefore, scoring or scaling the various welfare hazards would have added an unnecessary complexity to the audit without necessarily providing any material benefit to dog welfare. As with any welfare assessment, this audit relies on proxy measures of an animal’s welfare state if we accept that welfare is about how the animal feels [35], we also have to accept that any indirect measurement of these feelings will necessarily have the potential for error. The audit has attempted to incorporate multiple behaviours that may be rooted in similar negative affective states—for example, aggression and escape behaviour may well be rooted in anxiety/fear behaviour but by separating out these behavioural indicators we are maximizing the opportunity for these negative states to be recognized and for mitigations to be placed at the CCPs at which they occur.

In both field trials, the proportion of dogs observed showing signs of post-operative pain was similar, despite the administration of an analgesic drug in the peri-operative period. Factors influencing pain may include individual dog responses, surgical technique, tissue damage, and the post-operative environment [36,37,38,39]. Pain was recognised as a significant welfare problem but it can be minimised through the use of appropriate multimodal analgesia, such as the use of local anaesthesia alongside parenteral drugs, the employment of Halstead’s principles of surgery by a competent veterinary surgeon, and adequate post-operative nursing care and ongoing analgesic provision [11,36,39]. However, these preventative and remedial steps can only be taken if the pain is first recognised. Additionally, other behavioural signs of negative welfare may be even more challenging to identify. Inter-observer reliability testing showed that the agreements for ‘aggression’, ‘fear’ and ‘pain’ were reduced when the observer team was increased from two to three observers for field trial B. Whilst the agreements remained at levels that were within the bounds of acceptability, these results highlight the challenges of consistently recognising behavioural indicators of poor welfare, even amongst individuals experienced in dog behaviour. One reason for this may be the confound between pain and fear behavior, which has been highlighted in free-roaming dogs [40], therefore, it is important that observers note pain-specific aspects of behaviour such as wound interference and orbital tightening, and not just general muscle tension when evaluating different emotional states. Further research is needed to establish specific pain indicators in free-roaming dogs.

Regardless of such challenges, animal-based indicators have been suggested as valid proxies for negative emotional states in dogs [32] and as such, we would recommend that rather than discarding these indicators as being difficult to interpret, resources be focused on training staff who handle and interact with dogs in recognizing these behavioural indicators. In situations where recognising behaviours indicating negative welfare states is challenging, efforts should be made to ensure that veterinary surgeons of adequate skill levels are employed and ongoing multimodal analgesics are provided.

A direct comparison between CNR projects may be unhelpful, as each project works under different circumstances and with access to different resources, however, the welfare audit enables CNR projects to ‘benchmark’ their own progress in improving dog welfare by giving them a means of recording the prevalence of CCPs. Repeating the audit at a later time enables a CNR project to compare its progress against its own previous audit scores and identify whether the remedial action against CCPs has been successful in positively impacting dog welfare. In order to facilitate this process, a free mobile phone ‘Dog Welfare Assessment’ app has been developed for both Apple and android platforms [33].

The audit also allows for funding bodies to identify ‘good-practice’ standards. For example, the lack of a person suitably experienced in anaesthetic monitoring meant that 17%–22% of dogs observed under anaesthesia displayed signs consistent with their plane of anaesthesia being unsuitable for surgery, e.g., spontaneous head movements, jaw movements, vocalisations, etc. By comparing similar numbers of dogs in projects with and without a person suitably experienced in anaesthetic monitoring, it would be possible to evaluate the impact of a person suitably experienced in anaesthetic monitoring on the proportion of dogs that display signs consistent with their plane of anaesthesia being unsuitable for surgery, and thus make recommendations as to the importance of such a person to dog welfare. This type of assessment might be helpful in determining priorities when funding bodies allocate resources to CNR projects.

Some distinct differences in welfare issues were highlighted between the two projects. For example, Project A focused heavily on surgical and veterinary standards, using only Royal college of veterinary surgeons (RCVS) or equivalently qualified vets with a minimum of two years veterinary experience, which resulted in good standards of surgical asepsis, but moderate or severe welfare hazards were identified in the dog capture and handling stages of the project where the staff were less well trained. Conversely, Project B had invested heavily in staff training on dog handling but employed locally trained vets who may have had insufficient practical skills [11], thus, the welfare issues identified there were clustered within the peri-operative period.

The field trials highlighted welfare problems within the programmes consistent with deficiencies in training highlighted in earlier research [28]—namely the recognition and management of pain and other behavioural indicators of negative welfare, as well as highlighting specific welfare hazards, such as a lack of aseptic technique, or the release of dogs prior to food/water consumption, which may be an issue within some programmes but not in others. 

## 5. Conclusions

Catch Neuter Return programmes can provide a humane solution in dog population management, but it is important to recognise that the potential for dogs to experience negative welfare through the CNR process appears significant. Steps should be taken by CNR initiatives to ensure that staff are properly trained and resourced in order to ensure that they are able to recognise and mitigate negative dog welfare experiences. In particular, attention should be paid to measuring the welfare impacts on individual dogs experiencing CNR interventions, and not just on documenting the numbers of dogs neutered by a programme. This composite canine welfare audit is a practical, economical and robust tool, supporting CNR programmes in identifying and addressing potential welfare problems within their projects.

## Figures and Tables

**Table 1:** The Hazard Analysis and Critical Control Point (HACCP) process as applied to this study.

HACCP Steps	Project Application
1. Assemble the HACCP Team	Animal welfare research team at the University of Edinburgh
2. Describe Product	A robust composite canine welfare assessment tool that is practical for use within the CNR environment
3. Identify Intended use	To assess individual dog welfare in surgical CNR programmes
4. Construct a flow diagram to identify all stages of the catch-neuter-return (CNR) process at which CCPs may arise	Five stages of CNR identified (capture and transport, caging, peri-operative, post-operative and release) where CCPs may arise
5. List all potential hazards, conduct hazard analysis, consider control measures	Via a Delphi analysis using experienced stakeholders
6. On site confirmation of CNR stages flow diagram and potential welfare hazards	Visit to an existing CNR programme to confirm CNR process and trial of top-ranking suggested indicators from Delphi analysis as markers of poor welfare at an existing CNR programme
7. Determine CCPs	Refinement of welfare indicator list as per CCP algorithm. Ensure that all CCPs can be reliably identified
8. Establish critical limits for each CCP	Present or absent as all CCPs identified have a moderate-severe welfare impact on the individual, thus identification of any CCP is a welfare problem
9. Establish corrective action	Guidance on improving welfare
10. Establish verification procedures	Visit to a second CNR programme to validate selected indicators
11. Establish documentation and record keeping	Guidance notes on implementation of the welfare audit

**Table 2:** Comparison of the primary characteristics of two CNR projects used as field trial sites, highlighting the variations in the two projects.

Region	Field Trial Tite A	Field Trial Site B
	Africa	Asia
Number of dogs neutered/day	7–15	12–30
Capture method	Community handover, manual restraint, leashing	Manual capture, leashing, nets
Pre-surgical holding time	From one day prior	Same day
Veterinary team	Experienced veterinarians qualified in UK, USA, South Africa, Australia or equivalents. Local veterinary assistants	Locally trained veterinarians and veterinary assistants
Post-surgical holding time	Same day return	Next day return

**Table 3:** Results of the inter-attribute agreement analysis using Fleiss’ Kappa, of inter-observer reliability trial undertaken prior to field trial B.

Attribute	Response	Inter-Observer K	*p*-Value	Agreement
Demeanours	Aggression	0.845	0.00	Nearly perfect
	Escape	1.000	0.00	Nearly perfect
	Exploratory	1.000	0.00	Nearly perfect
	Fear	0.792	0.00	Substantial
Pain	Yes	0.864	0.00	Nearly perfect
	No	0.864	0.00	Nearly perfect

**Table 4:** Final welfare audit structure and valence of each CCP as a positive or negative welfare indicator.

Critical Control Point	Definition	Valence
**Program**		
Provision of DHPPi vaccination	Each dog entering the CNR programme receives DHPPi vaccination	+
Annual mortality rate >1%	Programme records are kept and the annual perioperative mortality rate is recorded as >1% (excluding euthanasias on humane grounds)	−
Facilities for treating additional medical conditions	Dogs presenting with additional medical or surgical needs are appropriately treated or humanely euthanased	+
Crash kit and drugs available	Kit should include appropriate drugs and quick reference dose charts in addition to CPR protocols	+
Biosecurity/hygiene protocol in place	A recognised process for cleaning all common areas should be in place on at least a daily basis	+
Positive community feedback on dogs	Human residents demonstrate positive verbal and physical interactions with dogs, and are supportive of the project	+
**Capture and Transport**		
Presence of injury due to capture/transport	Dog shows signs of wounding or injury after capture/transport	−
Mortality at capture/transport	Dog is dead after capture/transport	−
Use of capture equipment	Neck graspers, catch poles, or nets are used to capture the dog.	−
Blood on equipment	Blood on capture equipment indicating injury to at least one dog	−
Space to stand and lie comfortably in transport	Each dog is able to stand and lie down comfortably in the transport vehicle	+
Defecation/urination	Dog defecates or urinates on capture	−
Escape behaviour	Dog runs away from handler	−
Fear	Ear tension: ears down, often tucked back against head, tail tucked: tail tucked under hindlimbs, gaze aversion: won’t look directly at observer but turns or ducks head, whites of eye: can see sclera around eye, front paw lift: raising of one forepaw at a time	−
Aggression to catcher/handler	Baring of teeth, narrowing of eyes, raising of the hairs on the neck and back, shifting of weight to allow escape, growling, snarling and barking or snapping/biting directed towards the human handler	−
Inter-dog aggression	Baring of teeth, narrowing of eyes, raising of the hairs on the neck and back, shifting of weight to allow escape, growling, snarling and barking or snapping/biting directed towards another dog	−
**Cage/holding area indicators**		
Physical injury	Dog shows signs of wounding or injury that were not present upon capture	−
Inter-dog aggression	Baring of teeth, narrowing of eyes, raising of the hairs on the neck and back, shifting of weight to allow escape, growling, snarling and barking or snapping/biting directed towards another dog	−
Drinking water	Easily accessible potable water is provided to the dog in sufficient quantity	+
Bedding material	Dogs are provided with rubber matting, cardboard, newspaper, fabric or similar absorbent material	+
Signs of disease	Dogs exhibit signs of infectious disease e.g., nasal or ocular discharge, vomiting, pyrexia etc.	−
Vocalisation	Dog demonstrates repeated barking or howling behaviour indicating distress at confinement	−
Escape behaviour	Dog exhibits tunnelling, digging, wall bouncing, or biting behaviour towards cage/enclosure barriers	−
Fear	Ear tension: ears down, often tucked back against head, tail tucked: tail tucked under hindlimbs, gaze aversion: won’t look directly at observer but turns or ducks head, whites of eye: can see sclera around eye, front paw lift: raising of one forepaw at a time	−
Aggression to handler	Baring of teeth, narrowing of eyes, raising of the hairs on the neck and back, shifting of weight to allow escape, growling, snarling and barking or snapping/biting directed towards the human handler	−
**Peri-operative**		
Handler tests dog	Prior to handling dog, the human handler evaluates dog’s response to the human e.g., by slowly moving closer to dog, crouching and offering a closed fist to sniff, or stroking the dog	+
Aggression shown to handler	Baring of teeth, narrowing of eyes, raising of the hairs on the neck and back, shifting of weight to allow escape, growling, snarling and barking or snapping/biting directed towards the human handler	−
Dog injured during handling	Dog shows signs of wounding or injury not present prior to handling	−
Analgesic drug administration	Dog receives oral or parenteral administration of a recognised analgesic e.g., a non-steroidal anti-inflammatory drug, opioid, tramadol, and/or local anaesthestic infiltration.	+
Peri-operative mortality	Dog dies after anaesthesia is administered and prior to recovery from anaesthesia	−
Vocalisation during surgery	Dog emits audible noise during surgical procedure	−
Movement of head or forelimbs during surgery	Head, eyes, jaw or forelimbs exhibit spontaneous movement, indicative of consciousness	−
Break in aseptic technique	Surgeon, surgical instruments or surgical area is inappropriately prepared, or contaminated during the surgical process	−
Body condition score 1/3	On a scale of 1–3 where 1= emaciation and 3 = overweight, the dog scores a 1. Defined as “Bones easily visible (i.e., ribs, pelvis, lumbar vertebrae); loss of muscle mass, obvious waist and abdominal tuck” [29]	−
Surgery performed by student or untrained vet	Surgery performed by a non-qualified person e.g., a veterinary student or a vet untrained in CNR surgical procedures	−
Dedicated anaesthetic monitoring person	A person actively monitoring anaesthetic parameters e.g., heart rate, respiratory rate, reflexes etc., and trained to administer the correct dose of top-up anaesthesia under veterinary direction in order to maintain a surgical plane of anaesthesia	+
Excessive surgical time	Duration of surgery from initial incision to complete closure of incision >90 min	−
Ear tag placed	Ear tag placed in pinna as post-operative identification of neutering	−
**Post-operative**		
Drinking water	Easily accessible potable water is provided to the dog in sufficient quantity	+
Post-operative analgesia	Dog receives oral or parenteral administration of a recognised analgesic e.g., a non-steroidal anti-inflammatory drug, opioid, or tramadol.	+
Mortality	Dog dies after recovery from anaesthesia and prior to release	−
Individual post-operative assessment	Each dog is evaluated post-operatively for signs of pain, infection or other problems	+
Bedding material	Dogs are provided with rubber matting, cardboard, newspaper, fabric or similar absorbent material	+
Poor quality recovery	Dog exhibits staggering, disorientation, or a long recovery period	−
Pain behaviour	Vocalising, looking at or interfering with incision, hunched/tense posture, hunched/tense movement, reluctance to move, facial tension/ears back/eyes squinting, unresponsive/uninterested in interactions, nervous anxious or fearful	−
Escape behaviour	Dog exhibits tunnelling, digging, wall bouncing, or biting behaviour towards cage/enclosure barriers	−
Fear	Ear tension: ears down, often tucked back against head, tail tucked: tail tucked under hindlimbs, gaze aversion: won’t look directly at observer but turns or ducks head, whites of eye: can see sclera around eye, front paw lift: raising of one forepaw at a time	−
Aggression shown to handler	Baring of teeth, narrowing of eyes, raising of the hairs on the neck and back, shifting of weight to allow escape, growling, snarling and barking or snapping/biting directed towards the human handler	−
Inter-dog aggression	Baring of teeth, narrowing of eyes, raising of the hairs on the neck and back, shifting of weight to allow escape, growling, snarling and barking or snapping/biting directed towards another dog	−
Injury	Dog shows signs of wounding or injury (except for surgical incision)	−
**Release**		
Released prior to gaining full motor control or alertness	Dog exhibits unsteady gait, drowsiness or disorientation when released	−
Individual assessment prior to release	Dog is evaluated for pain, infection, hydration and level of consciousness prior to release	+
Reduced activity/physical impairment	Dog exhibits reduced activity, reluctance to move, abnormal gait or hunched or tense posture	−
Released in a different location	Dog is released in a location different from where it was captured	−
Presence of post-operative complication	Surgical incision shows signs of swelling, redness discharge or breakdown. Dog demonstrates an impairment related to clinical treatment e.g., injection site pain or infection	−
Released prior to food offered	Dog is NOT offered palatable, appropriate food after recovery from surgery and prior to release	−
Released prior to water offered	Easily accessible potable water is NOT provided to the dog in sufficient quantity after recovery from surgery and prior to release	−
Use of capture equipment	Neck graspers, catch poles, or nets are used to capture the dog.	−
Blood on equipment	Blood on capture equipment indicating injury to at least one dog	−
Fear	Ear tension: ears down, often tucked back against head, tail tucked: tail tucked under hindlimbs, gaze aversion: won’t look directly at observer but turns or ducks head, whites of eye: can see sclera around eye, front paw lift: raising of one forepaw at a time	−
Aggression shown to handler	Baring of teeth, narrowing of eyes, raising of the hairs on the neck and back, shifting of weight to allow escape, growling, snarling and barking or snapping/biting directed towards the human handler	−
Inter-dog aggression	Baring of teeth, narrowing of eyes, raising of the hairs on the neck and back, shifting of weight to allow escape, growling, snarling and barking or snapping/biting directed towards another dog	−
	Dog shows signs of wounding or injury (except for surgical incision)	−

**Table 5:** Relative prevalence of selected CCPs as observed at the two CNR projects ‘A’ and ‘B’.

Valence	Critical Control Point	CNR Project A (%)	CNR Project B (%)
+	Staff trained in dog capture	0	100
−	Vocalisation or movement of jaw or forelimbs during surgery	22	17
−	Break in aseptic technique during surgery	0	100
−	Poor quality recovery	17	18
−	Post-operative pain behaviour	56	60
−	Released prior to consumption of food/water	43	0
+	Individual assessment prior to release	0	17

## Appendix A

**Table A1:** Table of 53 dog welfare indicators and hazards generated by the literature review and Delphi process with an MRS > 30.

**Capture Indicators**		**MRS%**
1	Presence of injury due to capture	95.6
2	Lack of trained staff	32.6
3	Extreme ambient temperature (respiratory alkalosis and hypocapnia at ambient temperatures > 21 °C, elevated cortisol once core> 43 C)	65.2
4	Mortality	65.2
5	Blood on equipment	95.6
6	Barking/growling/aggression to handler	78.3
7	Increasing Chase-Capture time	76.1
8	Use of capture equipment e.g., nets, lasso (as opposed to manual handling)	65.2
9	Escape behaviour	60.1
10	Elevated respiratory rate	43.5
11	Defecation/urination	41.3
**Kennelling/cage indicators**		
12	Physical injury	84.8
13	Lack of biosecurity/hygiene protocol	43.5
14	Inter-dog aggression	30.4
15	Absence of drinking water	84.8
16	Escape behaviour e.g., bar biting, tunnelling	76.1
17	Absence of bedding material	73.9
18	Vocalisation/barking/howling	65.2
**Peri-operative indicators**		
19	Absence of analgesic drug administration	89.1
20	Aggressive restraint	37.0
21	Peri-operative mortality	37.0
22	Vocalisation/movement during surgery	93.5
23	Break in aseptic technique	65.2
24	Increased recovery time/poor quality recovery	32.6
25	Emaciated at time of surgery	30.4
26	Absence of crash kit	30.4
27	Use of unqualified veterinary personnel or students for surgery	30.4
28	Absence of dedicated anaesthetic monitoring person	70.0
29	Aggression	65.2
30	Decreased Heart rate variability (Increased sympathetic tone)	65.2
31	Increasing surgical time	65.2
32	Ear tag (4/7)	57.1
**Post-operative indicators**		
33	Vocalisations (crying, whimpering, groaning, screaming).	100.0
34	Pain behaviours (hunching, abdominal guarding, restlessness, altered facial expression, altered posture, increased body tension or flinching in response to gentle palpation of injured area and palpation of regions likely to be painful, rigid)	100.0
35	Absence of drinking water	91.3
36	Absence of post-operative analgesia provision	95.6
37	Attention to wound area (chewing, licking, looking, rubbing)	89.1
38	Absence of post-operative assessment	87.0
39	Absence of bedding material	73.9
40	Noticeable behavioural change compared to pre-operatively	84.8
41	Reduced exploratory and communicative behaviours	76.1
42	Human avoidance/fear behaviour	70.0
43	Escape behaviours	63.0
44	Elevated/ decreased nasal planum temperature	41.3
45	Mortality > 2%	65.2
46	Lack of biosecurity	30.4
47	Lack of staff to deliver care/monitoring	30.4
**Release indicators**		
48	Released prior to regaining full motor control or alertness	87.0
49	Reduced activity/physical impairment	67.4
50	Released in different location	39.1
51	Presence of post-op complications	32.6
52	Released prior to consumption of food and water	60.1
53	Released according to standard time schedule rather than individual assessment	80.4
